# When to Initiate Disease-Modifying Drugs for Relapsing Remitting Multiple Sclerosis in Adults?

**DOI:** 10.1155/2011/724871

**Published:** 2011-05-17

**Authors:** Mona Alkhawajah, Joel Oger

**Affiliations:** Multiple Sclerosis Clinic and Neuro-Immunology Laboratory, Department of Medicine, University of British Columbia, Room S-159, 2211 Wesbrook Mall, Vancouver, BC, Canada V6T 2H5

## Abstract

For patients with Relapsing Remitting Multiple Scierosis Beta Interfaerons and Glatiramer Acetate were the first to be licensed for treatment. This review deals with one major question: when to initiate therapy? Through exploring the unique characteristics of the disease and treatement we suggest an approach that should be helpful in the process of decision-making.

## 1. Introduction

Until the early nineties, the major challenge for Multiple Sclerosis (MS) neurologists was to establish the diagnosis when a patient presents clinical picture suggestive of MS. Treating MS patients consisted mainly of education, supportive measures, and symptomatic treatments. MS appeared as an unmodifiable illness with many stories of disappointment when using immunosuppressant therapy. When beta-Interferons (IFNs) and Glatiramer Acetate (GA) were introduced, and vigorously marketed, into the world of MS, patients' and neurologists' prospective about MS changed and focus in management shifted. Now, experts in MS around the globe strive to best answer the question that appears with every newly diagnosed patient: when to start treatment with Disease-Modifying Drugs (DMDs)? Which drug to use? 

This review deals with the former question. We will address the second question in a later review. Despite the absence of class I recommendations, we support the worldwide trend towards early treatment. We explore the possible reasons behind this consensus. We also address the implications of starting treatment early, and how one could explain the reluctance of some MS patients to start DMDs despite these medications being partly or completely supported by health benefits in numerous countries. We review these treatments in Relapsing Remitting MS (RRMS), since as we discuss in this paper, IFNs and GA were licensed for this disease phenotype. Efficacy was not shown for Primary Progressive MS (PPMS). We conclude by detailing our approach to the question of when to start, and we emphasize the importance of individualizing this decision to the patient's circumstances. 

In this review we focus on IFNs and GA as they are first-line treatments and widely available. We will not review in detail when to start Mitoxantrone or Natalizumab. Suffice it to say that we exceptionally use them as first-line when Relapsing Remitting MS (RRMS) appears to be rapidly leading to disability. Fingolimod has not been commercialized long enough that its place in the armamentarium against MS can yet be determined.

## 2. When to Start DMDs? Why Is It a Matter of Debate?

### 2.1. Characteristics of the Disease

In this section we discuss the features of MS that makes it a challenging disease. MS is mysterious for its true onset in individual patients, and thus talking about early versus late treatment remains to be a relative rather than an absolute argument. Here, we start by briefly exploring the difficulties that may face a neurologist diagnosing MS and prognosticating its path in individual patients.

#### 2.1.1. The Complexity of Making a Diagnosis of MS

Although patients may present with a highly suggestive clinical picture (typical age group and symptoms) supported by a brain MRI that fulfill the widely accepted Barkhof's criteria [1], many times the diagnosis is not straight forward. In the absence of a perfect marker, the diagnosis of MS should only be made after fulfilling the criteria of dissemination in place and time (McDonald Criteria) [2], and actively ruling out mimickers. It follows that delays in initiating treatment may result from waiting for a second clinical attack. The introduction of MRI to the routine practice of MS neurology paved the road for a new concept, Clinically Isolated Syndrome (CIS), or “first demyelinating event suggestive of MS”. With the ability to actually “see” the disease before it fully manifest clinically the concept of treating before fulfilling diagnostic criteria emerged. One problem is that although an abnormal MRI places a patient with a CIS at high risk to develop further attacks, there will remain a number of patients who will never develop MS. Racing with time, some neurologists will repeat the MRI after a few months, as MRI is known to be more sensitive to disease activity than clinical relapses [3]. This could hasten the establishment of the diagnosis and allow a more confident approach to early treatment.

#### 2.1.2. Prognosis of MS Cannot Be Predicted in Individual Patients

One of the most puzzling aspects of MS is its great variability in terms of natural course. A considerable percentage of 15–20% of MS patients will continue to mobilize freely and live independently throughout their lives without treatment [4]. The great challenge is to be able to tell who will and who will not run a benign course. Eighty percent of patients who present with a clinically isolated syndrome (CIS) and who have abnormal MRI brain, will convert to clinically definite MS (CDMS), and most do so within the two years of initial presentation [5]. MRI brain has proved to be so far the most useful tool in determining the likelihood of conversion in this clinical scenario. However it remains impossible to predict disease progression on the long-term at this stage, and may remains so even after 10 years from disease onset [6]. 

Most recent studies, however, suggest that the disease course during the first few years after clinical onset, and before the Expanded Disability Status Scale (EDSS) reaches a score of 3 to 4, is the most relevant in terms of predicting disability progression. In their natural history study of 806 essentially untreated MS patients, Scalfari et al. [7] showed that there was a correlation between the frequency of relapses during the first two years of the relapsing phase of the disease and the time to development of EDSS scores 6, 8, and 10. This correlation was absent for relapse frequency at any time after two years from clinical onset (defined as the date of the first symptom). These results came in accordance with those of another recent study by Leray and colleagues [8]. These studies agree with earlier ones of Confavreux et al. and Debouverie et al. [9, 10] demonstrating that the number of relapses was no longer a predictor of future disability once an EDSS score of 4 was reached.

 On the other hand, there remains no consensus on the definition of benign MS. While it is usually defined based on a long duration (longer than 15 years) and a low score of EDSS (no cutoff point has been agreed upon yet), it is important to keep in mind that EDSS scoring system does not parallel functionality, productivity, or quality of life. EDSS does not account for highly prevalent and disabling symptoms in MS like fatigue, depression, cognitive decline, and pain [11].

In an attempt to better understand benign MS and to reach a better definition for this disease phenotype, Rovaris et al. [12] reviewed the differences in MRI findings between two groups of RRMS patients, those who fulfilled the current definition of benign MS according to disease duration and EDDS score and those who did not. There were no differences between the two groups in the number of T2 lesions. However MRI brain of patients with benign MS showed relative sparing of eloquent areas, relative sparing of the cortex and of the infratentorial structures, less severe damage inside the macroscopic lesions and less diffuse damage outside these lesions. Interestingly functional MRI (fMRI) studies showed altered patterns of cortical activation and network connectivity in benign MS that may represent a compensatory mechanism through cortical reorganization. The authors explained how these differences may help in understanding benign MS and emphasized the importance of excluding cognitive decline when defining this phenotype, since there was evidence of more gray matter damage shown by magnetization transfer and fMRI in patients with benign MS and cognitive decline [13].

#### 2.1.3. What Is “Early” in MS

An important fact in MS is that the disease process starts several years prior to clinical presentation, if not very early in life. The epidemiological studies, particularly on the migrant populations, strongly suggest that a number of environmental factors may start their effect on genetically susceptible individuals very early in life, if not before birth [14]. 

 This proposal is amplified by the finding of a high percentage of abnormal conventional brain MRIs in patients with CIS who convert to CDMS [15, 16], the discovery of abnormalities in visual evoked potentials in CIS patients with and without history of optic neuritis [17], and the presence of cognitive decline in the very early stages of MS in patients with minimal locomotor disability and even in patients with CIS [18–20]. In addition, it is quite frequent that CIS patients will later recall past events that might have represented first clinical manifestation of MS. An interesting case report by McDonnell et al. describes a patient who clinically manifested PPMS a whole 10 years after an abnormal MRI brain. This patient served as a “normal control” in a study on Parkinson's disease when he had the MRI brain study [21].

Advanced MRI studies reveal several changes at the very earliest stages of MS or in patients with CIS undetected by conventional MRI [22–31]. Even before T2 Lesions appear, a special appearance of the white matter has been described: the dirty appearing white matter [32].

#### 2.1.4. When Is “Too Late” in MS

Sometimes one encounters patients years after the clinical onset of the disease, and the question of starting a DMD at this stage can be equally challenging. Two questions to consider here. The first is, would DMDs retain their efficacy even when started later during the relapsing remitting stage of MS? In other words, would the therapeutic window for DMDs within the relapsing remitting stage of the disease remain open as long as there are relapses and/or Gadolinium enhancing lesions, and probably closes when the progressive phase (without relapses) is reached? As clinical studies proved IFNs and GA to be much less effective in secondary progressive MS (SPMS) [33], the second question would then be, when would a patient be doomed to enter the secondary progressive stage? 

Most MS patients included in the pivotal IFNs and GA trials had a disease duration of one year or more; in one study disease duration of 6 months was allowed and in another, disease duration had to be of more than 3 years. Otherwise, disease duration was not an exclusion criterion except in one study when patients were excluded if they had MS for more than 15 years. Patients however were excluded if they had an EDSS score of more than 5.5 or 6, and in one study when EDSS was more than 3.5 [34–36]. On the other hand, in the BENEFIT trial on patients with clinically isolated syndrome (CIS), it has been demonstrated that patients who started off in the treatment arm did better than those who were crossed over to the treatment arm [37]. That was also demonstrated for RRMS patients included in PRISMS cross-over study [38], and in the GA US-based study [39].

**Summary 2.1.4**

A diagnosis of MS has to be made cautiously for its life-long implications. It is a stigma in the community and affects every aspect of life, including personal relationships, employment, and medical insurance. Tools for forecasting prognosis are lacking, and the natural course of illness is very variable. Disease behavior during the first two years and MRI changes early in the disease hold promise as ways of speculating the course of MS.

In order to tell about the true onset of MS, brain MRIs may need to be done on at-risk populations at a very young age (e.g., offspring of affected parents in areas of high prevalence of MS), however may remain very difficult to know without a biological or genetic marker for MS.

### 2.2. Characteristics of the Treatment (IFNs and GA)

Until recently, IFNs and GA were the only approved treatment for MS and remain the best studied to date. Despite the growing trend towards earlier treatment, DMDs will not be always instituted as soon as a diagnosis is made, or anticipated as in CIS. In order to understand why, we review the effects and limitations of the currently available DMDs, their safety, and the evidence supporting starting treatment after a CIS in the following section.

#### 2.2.1. DMDs in MS: Effectiveness and Cost-Effectiveness

**(1) Effects on Relapses and Conversion to SPMS**

In randomised clinical trials (RCTs) efficacy of DMDs in RRMS was measured based on their effect on relapses frequency and severity, effect on MRI parameters (number of new T2 lesions, T2 lesions load, lesions enhancement, brain atrophy, etc.), and effect on confirmed changes in EDSS. In studies on patients with CIS the primary focus was the time to conversion to CDMS.

The pivotal studies that lead to licensing of IFNs and GA were heterogeneous to some extent and difficult to compare. Each had its own strengths and limitations. In the literature there is a considerable number of reviews and meta-analyses, interestingly with variable and sometimes conflicting conclusions, depending on the weight given by each author or reviewer to certain points of weakness or limitation, the time when the analysis was done (before or after other studies or open label extensions were completed), and probably personal experience.

In general, one can say that all currently available IFN preparations and GA proved superior to placebo in terms of effect on frequency of relapses in RRMS patients. In the pivotal trials [40–44], they all reduced the frequency of relapses to a similar degree, almost 30% compared to placebo group [39, 44–46]. The protective effect of the IFNs and the GA on relapses was recognized during the first year of treatment. Critiques argued that this effect was no more present for GA and could not be confirmed for IFNs, during the second year. For IFNs, the reason was the high number of dropouts and the failure to do an intention-to-treat analysis [45].

On the other hand, open label studies for both agents, despite all the inherent limitations of this study design, suggest a sustained benefit for these DMDs on relapse frequency. It is however expected in MS patients to have fewer relapses as the disease duration increases, when degenerative changes override inflammatory events. It is also difficult to make absolute conclusions from these studies due to the fact that only a proportion of patients who were initially included continued to follow up. One may wonder if the patients who did not enter the follow up actually fared worse. Propensity to bias due to loss of randomization may render the trials less representative of the natural history of RRMS. Moreover, patients may have not been monitored, may have discontinued the drug for considerable amount of time, switched to another DMD, or added another one [47, 48].

In addition to the effect on relapse rate, open label extensions of the original studies and nonrandomised retrospective studies showed delayed time to conversion to SPMS and to reaching high EDSS scores in the treated group [47–50]. Although these nonrandomised prospective or retrospective studies carry less weight in evidence-based medicine because of the reasons discussed, the fact that they all go in the same direction would be taken as an evidence. However the energy of the pharmaceutical industry supporting them may deprive them of this very relative advantage.

**(2) Effects on MRI Parameters**

The effects of the IFNs and GA on MRI parameters in the pivotal trials in MS was even more robust than the clinical effect, and further supported their role in managing MS. Studies, including those which followed the pivotal trials, suggest sustained effects on diseases burden as seen on MRI. A favourable effect was demonstrated on the number of enhancing lesions, rate of black holes formation, as well as on the rate of brain atrophy [51–54]. Importantly, an inverse correlation was found between T1 hypointense and T2 hyperintense lesions, and the absolute change in brain volume over 6 years [54], and there was a significant correlation between the changes in T1 hypointense lesions from baseline to year 2 and changes in supratentorial brain volume from year 2 to 6.

The relation between the rate of brain atrophy and the subsequent development of disability was well-demonstrated by Fisher et al. in an 8-year follow up study for the patients originally involved in a phase III IFN-1a trial [55]. In this study, the percent change of brain parenchymal fraction (BPF) over two years correlated with both EDSS and MS functional composite (MSFC) at 8 years. This makes the effect of DMDs on brain atrophy clinically relevant, and indicates that the rate of brain atrophy could be considered a surrogate marker for predicting disability progression. Interestingly, however, there was no difference in BPF and BPF percent change at follow up, between the group of patients who were originally randomised to receive treatment versus the group who was not. In other words there was no correlation between the BPF and the BPF percent change, and the total time on DMD from baseline to the 8-year follow up.

In a small open-label, single-blinded study, a positive effect of IFN-1a on the gray matter fraction (GMF) but not white matter fraction (WMF) after a follow up period of 3 years was suggested. This finding may indicate different dynamics and mechanisms behind white matter and gray matter atrophy in MS, and that the favorable DMD effect on brain atrophy might be mainly through reducing GMF atrophy [56]. The importance of gray matter atrophy and its correlation to disability particularly measured by MSFC was demonstrated by Rudick et al., who followed 70 patients and compared them to 17 healthy controls (HCs) over an average period of time of 6.6 years [57]. Based on the results of this study, the authors suggest that EDSS is less sensitive in more disabled patients with more brain atrophy than MSFC, and propose that MSFC correlates better with pathology than EDSS does. In another study, cervical cord atrophy was also found to be associated with disability measured by EDSS and MSFC, independently from gray matter atrophy [58].

**(3) The Ultimate Goal: Effects on Disability**

**(i) Disability as Measured by EDSS**

Although it is desirable to have less and milder relapses during the course of MS, the ultimate goal of DMDs therapy in MS remains to be preventing, or at least delaying, the progression of disability. A recent meta-analysis by Sormani et al. [59] showed that there is a significant correlation between the effect of treatment on relapse rate, emergence of new MRI lesions, and confirmed EDSS progression. This finding supports the use of EDSS as an endpoint in clinical trials in MS, particularly that there was a strong correlation between the EDSS changes during the first two years of randomization to a phase III IFN trial, and the likelihood of reaching clinically important EDSS scores 8 years after randomization [60].

**(ii) Cognitive Disability**

Another important aspect of measuring the efficacy of DMDs is through their effect on cognition and fatigue; two major sources of disability which are not captured by EDSS. An effect of DMDs on cognitive decline can be inferred from the fact that they may reduce brain atrophy, particularly their effect on gray matter, as discussed above. However it is certainly more convincing to look at the studies that compared DMDs-treated and untreated MS patients in relation to the degree of cognitive impairment.

Pliskin et al. [61] was among the first to study the effects of IFN-1b on cognition in MS patients. Delayed visual reproduction test performance improved in the subgroup of patients who were treated with high dose IFN-1b. The study was done on a total of 30 MS patients originally randomised to receive high dose, low dose, or no IFN-1b treatment, as part of the pivotal IFN-1b trial.

However, Montalban and Rio [62], through reviewing 6 studies on the effect of DMDs on cognition (1996–2003) including Pliskin's, concluded that the effects of DMDs on cognition is not clear and the results are inconclusive, and that more studies need to be conducted.

The only pivotal IFN study that incorporated neuropsychological testing was that for Avonex [63]. The results suggest significant effect of Avonex injections on cognitive function, particularly domains measured by PASAT. Only few studies were published more recently on the effect of the IFN preparations on cognition; none were randomised double-blinded placebo-controlled trials [64, 65]. Limited conclusions can be made out of the results of these studies.

Same holds true for GA. More than 10 years ago, Weinstein and colleagues [66] performed the first large-scale study on the effect of a DMD on cognitive function in MS. Cognitive performance rather improved in both the placebo and the treated group at 1 and 2 years follow up. Moreover, cognitive function at baseline was largely intact at baseline in both groups. The authors concluded that GA did not show a protective effect on cognition, and this could be due to a number of reasons, including lack of significant impairment at base line, short follow up period and lack of deterioration in both the treated and the control group, and insensitivity of the testing tools to changes occurring over two years in this population of patients. No studies dedicated to test the effect of GA on cognition were published thereafter, and it remains unclear if GA can prevent cognitive decline.

**(iii) Effects on Fatigue**

As we pointed out earlier in this review, fatigue is one of the most debilitating symptoms in MS, even in patients with low EDSS scores, and it affects quality of life and employment. A major problem with fatigue is that surrounding people with limited knowledge about MS may not appreciate the impact of this symptom on the ability to work. Fatigue is one major aspect of MS that could benefit from treatment with DMDs. A good number of studies looked at the correlation between brain atrophy and fatigue. The importance of this correlation relies in the fact that DMDs may actually prevent brain atrophy, as discussed above. From there one can infer that DMD may then reduce the incidence or severity of fatigue by reducing the rate or degree of brain atrophy. Studies, however, did not agree as whether or not fatigue can be correlated with MRI brain measures like lesion load or brain atrophy. Bakshi et al. [67], studied 71 consecutive RRMS and SPMS patients. They looked at correlations between fatigue as measured by the Fatigue Severity Scale, and some of the conventional MRI brain parameters including lesions load, degree of focal or global brain atrophy, and presence of lesions in the brainstem. They could not find a correlation between fatigue and these MRI parameters. This is in accordance with what Werf et al. found in his study of 45 patients with RRMS and SPMS [68].

On the other hand, a number of recently published studies did show an evidence for an association between brain atrophy in MRI and fatigue [69–72], and in some of them, it was mostly the gray matter atrophy that was found to be correlated, particularly the deep gray nuclei and the cortex of the parietal lobe.

There are no placebo-controlled studies on the effect of DMDs on fatigue in MS patients. In his observational study, Ziemssen et al. [73] found that GA treatment ameliorated fatigue and reduced the number of days taken off work, when a comparison was made between the 3 months prior to starting treatment and the last three months of follow up after initiating treatment (total observational period of 12 months). As the authors indicated there is a number of limitations to this study, including weaknesses inherent in this study design, particularly absence of a comparative group. In a cross-sectional study, the investigators could not demonstrate differences in fatigue prevalence or severity between treated and treated MS patients [74]. Treatment included a variety of DMDs and immunosuppressant medications.

**(4) Cost-Effectiveness**

There is a number of studies that dealt with the economic impact of multiple sclerosis by looking at the direct and indirect costs of the disease, and how do DMDs contribute to the total financial burden.

Directs costs of MS are the medical costs including payments made to healthcare providers for inpatients and outpatient care, physical therapy, rehabilitation, laboratory services, drugs, and so forth, Indirect costs are those related to loss of productivity and loss of Quality of Life (QOL).

It became obvious through the results of these studies that the more advanced the disease the higher are the costs, and that the indirect costs of the disease are the major contributor to the cost of illness in MS [75]. Indirect costs increase in proportion with the loss of QOL, and the latter seems to improve with Interferon treatment [76].

The evaluation of DMDs cost-effectiveness was for the major part done through cross-sectional study of direct costs, the so called Economic Modeling Studies. Cost-effectiveness was tested through measuring cost per quality adjusted year life (QALY). Studies were heterogeneous in many ways and difficult to compare. Parkin et al. [77] concluded that treatment with IFN-1b did not produce long-term gains in QALYs. So did Phillips in a review study [78].

Other economic studies reached different conclusions in regard to cost-effectiveness of treatment with IFNs, including for treatment after CIS [79, 80]. These studies were funded by the manufacturing companies.

The uncertainty around the cost-effectiveness of DMDs reflect the complexity of measuring this endpoint, and the high variability of study design prevented us from reaching a definite conclusion. Detournay [81] discusses the limitations and difficulties of the economic modeling studies in evaluating DMDs cost-effectiveness, not only in performing them, but also in understanding them.

#### 2.2.2. Safety of DMDs

IFNs and GA have been in use for about 20 years. They proved to be safe and well-tolerated.

Adverse events from the use of IFNs and GA may be divided in to three major categories.

1. Common but non life-threatening side effects, mainly flu-like symptoms with IFNs injection, and subcutaneous adipose tissue necrosis with GA injections. Despite being referred to as “benign side effects”, flu-like symptoms and injection site reactions are recognized as major factors in impaired adherence to treatment, as we discuss below.
2. Rare but serious side effects: fulminant liver failure and skin necrosis, with the use of IFNs and GA respectively.
3. The emergence of NAB during the course of treatment with IFNs.

A recently published cross-sectional study, although insensitive to rare side effects, proved long-term safety of IFN-1b for RRMS after a 16-year follow up [82]. In an open-label extension study of the phase III trial of intramuscular IFN-1a, patients who received treatment for 6-8 years were assessed for immunogenicity and safety [83]. Again the drug proved safe and well-tolerable. The rates of depression were found similar to those in the general MS populations. However dedicated studies looking at correlation between treatment with IFNs and depression in terms of incidence and severity need to be conducted before definite conclusions can be drawn. Miller et al. [49] evaluated a small number of patients who were treated with GA for a median duration of 12 years (range 1–22 years) in an open-label design. GA was shown to be safe and well-tolerable. No patients in this cohort of 46 developed skin necrosis. 

Two recent prospective studies on a small number of patients [84, 85] suggested that treatment with GA or IFNs might be safe during pregnancy. In one study [84] nine patients were exposed to GA for the whole period of pregnancy. However, there might be increased risk of miscarriage, spontaneous abortions, and low birth weight. Moreover it remains to be discovered the long-term developmental adverse events in exposed foetuses.

#### 2.2.3. Treatment after CIS

The current literature abounds in studies and analyses supporting the initiation of DMDs early in RRMS or even after the first demyelinating event suggestive of MS (CIS) [5, 9, 10, 33, 37, 59, 86–88].

This may represent a global trend [33, 89–93] towards earlier treatment in the hope of preventing irreversible damage. It may also represent the effects of relentless and vigorous efforts from the manufacturing companies, and probably as well patients and media pressure to “do something” for a young population with potentially disabling illness.

However, this trend is justified by the available evidence. As discussed above, the characteristics of the disease (MS) and the characteristics of the treatment (IFNs and GA) support the use of DMDs after the CIS. Goodin and Bates [5] list a number of facts in favour of a decision of treatment after CIS.

1. Evidence of axonal injury early on in the disease [94, 95]: axonal injury was present even in the absence of disability and progressed more rapidly during the earlier stages of the disease.
2. Evidence of brain atrophy at the earliest stages of the disease; higher rate of atrophy occurred in RRMS, compared to SPMS [55, 96–98].
3. In CIS patients, the number of lesions at baseline MRI, and the increase in lesion volume over the first 5 years, both correlated with EDSS at 14 years [99].
4. Treatment with IFNs or with GA delayed conversion to CDMS. In addition, larger treatment effects were obtained when treatment was started in the CIS stage than when started in the RRMS stage, and larger when started in the RRMS stage than when started in the SPMS [41, 42, 100–109].

**Summary 2.2.3**

Collectively, IFNs and GA are effective in modifying the natural course of MS through their partial, albeit significant, impact on relapse frequency, disease burden on MRI, and time to sustained disability. Their impact on other aspects on MS particularly disabling symptoms like fatigue and cognitive impairment remains to be confirmed. The inherently limited studies on cost-effectiveness support their use, and postmarketing surveillance proved their safety. The rationale behind starting treatment as early as after a CIS suggestive of MS and supported by abnormal MRI brain seems to be well-supported by a growing number of clinical and pathological studies, however other important considerations, as discussed below, argue against starting treatment after a CIS in every single patient, and thus starting early cannot be considered as a standard of care.

### 2.3. Implications of Starting Treatment Early 

#### 2.3.1. Long-Term Commitment and Adherence

MS is a chronic disorder, and it is not yet known when it is ideal to stop DMDs once started. Patients knowing this about their treatment may reduce their tolerance to what we, as physicians, identify as minor side effects, and may impair long-term adherence. Starting DMDs after a CIS could mean starting during early to mid-twenties, when a patient is busiest studying or working, with many plans and dreams on board. For women of child-bearing age, the decision to start a DMDs might well be affected by or affect their plans to conceive, and that should be taken in consideration too. 

Treatment discontinuation ranges from 14 to 44% in long-term postmarketing studies of IFNs [110]. The most common reasons for discontinuation were side effects (most commonly injection-site reactions and flu-like symptoms) and unrealistic expectations of IFNs treatment. Tremlett and Oger [111] found that the number one reason for interruption of IFNs therapy was perceived lack of efficacy. Twenty-five percent of patients taking DMDs (IFNs or GA) were found nonadherent to treatment, according to the results of the Global Adherent Project [112]. In this study, the two most common reasons for nonadherence were: forgetting to take the injections and injection site reactions.

#### 2.3.2. Neutralizing Antibodies (NABs) to IFNs

There is yet no global consensus on the clinical significance of the development of NABs in MS patients treated with IFNs. However a panel of European experts believe that NABs affect the clinical efficacy of IFNs, and they recommended a set of actions to be taken depending on how well the patient is doing clinically [113].

Many factors contribute to the emergence of NABs including the formula of the IFN used, route of administration, dose and frequency [114]. NABs do not persist in every patient, with patients with higher titers being more likely to have persistently positive testing to NABs.

Developing NABs early during the disease course, as in the case of starting treatment after a CIS, may have different effects on treatment success than when NABs develop later. Indeed this has not being studied before, but is worth considering when deciding about initiating treatment early.

#### 2.3.3. Participation in Ongoing or Future Clinical Trials

Being on one of the current DMDs may deprive the patient from the chance to participate in clinical trials testing new treatments in MS, or may delay his or her entry to the study. It also means that it will be more difficult to form a control group to which treated patients can be compared prospectively or retrospectively.

### 2.4. How to Answer the Question “When” in the Clinic

In a patient with a single demyelinating event suggestive of MS, the decision of whether to treat or not and when to start a DMD has to be made by mutual agreement between the treating neurologist and his/her patient. In order to optimize adherence and achieve maximum benefit from treatment, a patient must be well-informed, contribute to decision making, be comfortable with the decisions made, and be willing to commit. 

#### 2.4.1. Acknowledge Patients' Differences in Attitude towards Treatment

Ross [115] discussed the importance of knowing what attitude does a patient hold towards treatment. There are 4 groups of patients: treatment-believers, treatment-suspicious, treatment-seekers, and treatment-disheartened. Discussion has to be tailored accordingly. Because adherence is a goal in treatment, the answer to when to start a DMD may vary between these groups of patients, even for those with very similar disease characteristics.

#### 2.4.2. Patients Need to Be “Well Informed”

The patients should be made aware of the limitations of the current treatment options. Realistic goals need to be set, and the patients should learn that this is not a “symptomatic” treatment. Having relapses does not necessarily indicate treatment failure. Side effects need to be addressed and strategies to decrease their frequency, intensity, and impact on their lives should be taught. Patients' personal circumstances may prevent them from starting treatment at a certain point of time, when they cannot handle extra stress in their lives. For the sake of optimizing adherence, it could be wise therefore to wait till after patients are more capable of handling all the difficulties around starting a new treatment. If the different companies have setup support services for MS patients starting on DMDs, it is our deep-felt conviction that these companies cannot do as good a job as the MS clinic nurse who already knows the patient, his/her personal circumtances, character and approach to life. This nurse should be the person in direct contact with the patient to help him/her to make the appropriate decision as to when and what to use as a DMD.

Patients will ask about newly emerging therapies. The effects on being on one of the current first-line DMD prior to starting treatment with one of the newer agent are not yet known. The possible impact on the response to treatment with the newer agent or on development of side effects with the newer treatment is not known as well.

#### 2.4.3. Local Policies and Healthcare System

Finally, in an ideal world, financial limitations should not play a major role when it comes to making decisions about treatment. However an ideal world does not exist. A neurologist's decision will be frequently influenced by the health care system regulations. Free medical care is not available everywhere. In certain areas, certain treatments are not available to every patient, depending on a number of factors including the type of insurance they hold, the rules and regulation of the country or province where they live, and the type of disease they have. The Helsinki accord which has not been signed by all DMD companies, call for trials not to take place in the countries where the drug will not be available for patients post trial. Unfortunately the number of these countries has been increasing rather than decreasing. We hope this will convince big Pharma to make the drugs more available rather than continue the race for the most expensive products.

## 3. Conclusion

After considering all the different factors discussed in this review, our answer to the question *When to Start Treatment* remains highly individualized. Our reading of the literature only partly supports early treatment after clinical onset of the multiple sclerosis. We do not think that delaying treatment by a few months after the onset of a CIS impacts the long-term prognosis.
